# Oscillatory neuronal dynamics associated with manual acupuncture: a magnetoencephalography study using beamforming analysis

**DOI:** 10.3389/fnhum.2012.00303

**Published:** 2012-11-16

**Authors:** Aziz U. R. Asghar, Robyn L. Johnson, William Woods, Gary G. R. Green, George Lewith, Hugh MacPherson

**Affiliations:** ^1^Hull York Medical School, University of HullHull, UK; ^2^York Neuroimaging Centre, University of York, The Biocentre, York Science ParkYork, UK; ^3^Department of Psychology, University of YorkYork, UK; ^4^Brain and Psychological Sciences Research Centre, Swinburne University of TechnologyVictoria, VIC, Australia; ^5^Faculty of Medicine, Department of Primary Care, University of SouthamptonSouthampton, UK; ^6^Department of Health Sciences, University of YorkYork, UK

**Keywords:** acupuncture, magnetoencephalography, beamforming, oscillations, somatosensory cortex

## Abstract

Magnetoencephalography (MEG) enables non-invasive recording of neuronal activity, with reconstruction methods providing estimates of underlying brain source locations and oscillatory dynamics from externally recorded neuromagnetic fields. The aim of our study was to use MEG to determine the effect of manual acupuncture on neuronal oscillatory dynamics. A major problem in MEG investigations of manual acupuncture is the absence of onset times for each needle manipulation. Given that beamforming (spatial filtering) analysis is not dependent upon stimulus-driven responses being phase-locked to stimulus onset, we postulated that beamforming could reveal source locations and induced changes in neuronal activity during manual acupuncture. In a beamformer analysis, a two-minute period of manual acupuncture needle manipulation delivered to the ipsilateral right LI-4 (*Hegu*) acupoint was contrasted with a two-minute baseline period. We considered oscillatory power changes in the theta (4–8 Hz), alpha (8–13 Hz), beta (13–30 Hz), and gamma (30–100 Hz) frequency bands. We found significant decreases in beta band power in the contralateral primary somatosensory cortex and superior frontal gyrus (SFG). In the ipsilateral cerebral hemisphere, we found significant power decreases in beta and gamma frequency bands in only the SFG. No significant power modulations were found in theta and alpha bands. Our results indicate that beamforming is a useful analytical tool to reconstruct underlying neuronal activity associated with manual acupuncture. Our main finding was of beta power decreases in primary somatosensory cortex and SFG, which opens up a line of future investigation regarding whether this contributes toward an underlying mechanism of acupuncture.

## Introduction

A number of human functional MRI (fMRI) investigations have been performed during manual insertive acupuncture needling to spatially map the areas of the brain which show significant alterations in activity (Hui et al., 2000, 2005, 2010; Dhond et al., 2007; MacPherson et al., 2008; Asghar et al., 2010). These fMRI studies, which utilize the blood oxygen level-dependent (BOLD) contrast to provide a cerebral haemodynamic measure and thereby an indirect indication of neuronal activity, have enabled substantial advances of our understanding of the effect of manual acupuncture needling in brain areas. For example, whole brain fMRI studies report changes in BOLD signal in a number of locations including the somatosensory cortex, sub-cortical and limbic areas, and in areas of the frontal lobe including the superior frontal gyrus (SFG) (Hui et al., 2000, 2005, 2010; Fang et al., 2004; Napadow et al., 2005; Dhond et al., 2007; MacPherson et al., 2008; Asghar et al., 2010).

Notwithstanding the usefulness of fMRI in studies of manual acupuncture, it is desirable to have non-invasive measures which are more directly representative of neuronal activity. Electroencephalography (EEG) records with a high temporal resolution the electrical potentials at the scalp surface generated from underlying neurons. The results of EEG spectral analysis with manual acupuncture have been variable with reports of no alterations in neuronal rhythms (Rosted et al., 2001), and modifications in the delta (Kim et al., 2008), theta (Kim et al., 2008; Hsu et al., 2011), alpha (Kim et al., 2008; Streitberger et al., 2008; Chang et al., 2009), and beta (Kim et al., 2008) frequency bands. There are major obstacles associated with accurate intracranial source reconstruction as the EEG signals recorded at the scalp surface have been distorted by various tissues including the meninges, cerebrospinal fluid and the skull. Magnetoencephalography (MEG) is a non-invasive imaging technique which detects the external weak neuromagnetic fields near to the head which are associated with underlying neuronal currents generated from brain sources. Since the extracranial magnetic fields remain unaffected by the intervening skull and scalp tissues, MEG neuroimaging has the advantage of enabling relatively undistorted estimates to be made of neuronal source localizations and oscillatory dynamics.

Experimental protocols in MEG neuroimaging investigations typically consist of many repetitive presentations of a particular stimulus such as visual, auditory, or somatosensory, and determine evoked responses (phased-locked to the presenting stimulus). The onset time for these types of sensory stimulus is measurable and referenced in relation to the recorded MEG signals. This subsequently allows the application of various analysis techniques such as dipole fitting, minimum norm, and spatial filtering methods to provide estimates of the underlying neuronal sources locations. Such a methodological and analysis approach has been taken advantage of in MEG studies of acupuncture using electroacupuncture or acupressure where the stimulus onset times are able to be measured since these stimuli are initiated electronically. The results of such investigations have revealed mappings onto the primary somatosensory cortex (Dhond et al., 2008; Witzel et al., 2011) with alterations in event related synchronizations/desynchronizations in various frequency bands (Witzel et al., 2011). In contrast to electroacupuncture or acupressure, when administrating manual insertive acupuncture the onset time for each needling stimulus is not discernible due to the inherent complex nature of the applied stimulus. We postulated that a potential way forward in overcoming this absence of stimulus onset timing with manual acupuncture needling manipulations was to exploit beamforming (spatial filtering), which is not reliant upon time and phased-locked stimulus driven neuronal responses (Hillebrand and Barnes, 2005; Hillebrand et al., 2005). The identification of such induced responses by the beamformer also enables measures to be made of the increase or decrease in cortical oscillatory power within selected frequency bands (Hillebrand and Barnes, 2005; Hillebrand et al., 2005).

In the current study, we performed a whole head beamformer analysis of MEG signals acquired during manual acupuncture needling at the right LI-4 (*Hegu*) acupoint to determine whether or not this could reveal any significant underlying neuronal localizations and oscillatory dynamics in the brain. Specifically, we contrasted epochs of manual acupuncture needle manipulation with epochs in the baseline (prior to needle insertion) period (*needle manipulation minus baseline* contrast) and determined areas of significant neuronal localization where there were modifications in power in the theta (4–8 Hz), alpha (8–13 Hz), beta (13–30 Hz), and gamma (30–100 Hz) frequency bands.

## Materials and methods

### Study participants

Thirteen right-handed adult volunteer participants (seven females, six males) who were naïve to acupuncture were recruited (mean age ± SD, 41 ± 16 years). All participants self reported that they had no neurological or psychiatric disorders. No financial reward was given to study participants. Written informed consent was obtained prior to participation in the study. Ethical and scientific approval for the investigation was sought and obtained from the Research Ethics and Governance Committee, York Neuroimaging Centre, University of York.

### Manual acupuncture needling

Manual acupuncture was performed at acupoint *Hegu* (LI-4) by an experienced acupuncturist (HM) during MEG scanning. The needles used were sterile, single use and manufactured from non-magnetic stainless steel (25 mm length, 0.28 mm diameter, Hwato, China). Needles were inserted into the first dorsal interosseous muscle of the right hand at either a depth of 8–12 mm or superficially at 1–2 mm as described previously (MacPherson et al., 2008; Asghar et al., 2010). Only data obtained using the 8–12 mm depth is considered in the current study.

The needling protocol during MEG scanning was as detailed in our previous neuroimaging of acupuncture investigations (MacPherson et al., 2008; Asghar et al., 2010). The acupuncturist received needling instructions on a projection screen. In brief, following a two-minute rest baseline period, an acupuncture needle was inserted at LI-4 in the right hand. After a two-minute post-needle insertion period, there was a two-minute period of needle “stimulation” using the “even” method (Deadman et al., 1998) composed of continuous alternating 180° rotations of the needle clockwise and anticlockwise at approximately two cycles per second (needle manipulations). Next, the needle manipulations were stopped for four minutes (needle remained inserted) followed by another two-minute period of needle rotations. The acupuncturist performed the needle insertion and needle manipulations without making any hand to hand contact with the participant. In our MEG analysis, we only considered the contrast between the first two-minute period of needle manipulation and the two-minute baseline period prior to needle insertion (*needle manipulation minus baseline* contrast).

### Magnetoencephalography scanning

MEG scanning was performed with participants in a comfortable supine position using a 4D Neuroimaging Magnes 3600 whole head system with 248 magnetometers in a magnetically shielded room. Participants were instructed to keep eyes closed throughout the duration of the scanning. The acquisition sample rate was 648.17 Hz, and data were online band passed filtered from 1 to 200 Hz. Five MEG coils (placed on the left and right pre-auricular locations and three equidistantly spaced on the forehead) provided landmark positions with reference to the MEG sensors and allowed monitoring of head position. Head movements values were <0.5 cm in all participants. The head shape of participants was 3D-digitized using a Polhemus stylus (Polhemus Fastrak) and was utilized for accurate co-registration with the structural MRI scan (Kozinska et al., 2001). After the MEG scan, a 3 Tesla GE Signa Excite HDx (General Electric, Milwaukee) MRI scanner with an eight channel head coil (GE Signa Excite 3.0T, High Resolution Brain Array, MRI Devices Corp., Gainesville) was used to obtain high resolution axial T1-weighted structural images of the whole head using an inversion recovery prepared 3D Fast Spoiled Gradient Echo pulse sequence (TR = 7.5 s, TE = 3 ms, flip angle 20°, acquisition matrix 256 × 224 interpolated to 512 × 512, field of view = 29 cm, in plane resolution 0.6 × 0.6 mm, slice thickness 1.0 mm).

### Beamforming source localization

We divided the two-minute baseline period, and the two-minute period of needle manipulation, into 120 consecutive 1 s epochs. Each 1 s epoch was visually inspected and those containing artifact contamination (movement, eye blinks, swallows, and electrical noise) were excluded from further analysis. The mean ± SD artifact-contaminated epochs rejected in the baseline period was 14 ± 7 epochs, which was not significantly different from the 23 ± 14 epochs rejected during the needle manipulation period (*P* > 0.05, Wilcoxon rank test).

Beamforming (adaptive spatial filtering) was performed using a vectorized linearly constrained minimum-variance beamformer technique (Van Veen et al., 1997; Huang et al., 2004) for all the artifact-free 1 s epochs for the baseline and needle manipulation periods, generating a neural activity index (a measure of total power) for both. Independent beamformers were constructed at each grid point using a 5 × 5 × 5 mm grid which covered the whole brain. After filtering the data within selected frequency bands and beamforming, the total power of the time series was calculated by squaring the value at each time point and summing together. The spatial filter provides an estimate of the underlying neuronal current source and the oscillatory dynamics at each grid location (Van Veen et al., 1997; Huang et al., 2004).

At the first level analysis, statistical *z*-value maps (obtained by transforming an unpaired *t*-value to normalize for degrees of freedom) of power increases and decreases for each individual were generated for the contrast *needle manipulation minus baseline* for data band pass filtered in the following selected frequency bands: theta (4–8 Hz), alpha (8–13 Hz), beta (13–30 Hz), and gamma (30–100 Hz). At the group level, a one-sided t-statistic map based on the mean and variance of the individual z-maps was generated. Non-parametric permutation statistics (Nichols and Holmes, 2002) was then utilized to generate probability maps of *t*-values with *P* < 0.01. Maximum statistics were used to correct for multiple comparisons using the single threshold test (Nichols and Holmes, 2002). The statistical significance testing was performed using a null distribution generated from a permutation procedure for voxels over the whole brain. In the group analysis, all participant's *t*-value maps were spatially normalized onto the Montreal Neurological Institute standardized brain (MNI152). As the whole brain MEG beamformer analysis showed significant power modifications only in the primary somatosensory cortex and the SFG brain areas, we delineated the borders of these areas using the Harvard-Oxford cortical probabilistic atlas (http://fsl.fmrib.ox.ac.uk/fsl/fslwiki/Atlases) with a relatively conservative probabilistic thresholding (≥30%).

In fMRI neuroimaging studies the number of significant voxels in a particular brain structure is commonly reported. However, due to the underlying beamformer spatial filter assumptions and design this could result in, for example, a wide spread of activity from a relatively weak source and a subsequent high voxel count. Instead, we preferred to calculate, following permutation statistical testing, the exceedance mass (Bullmore et al., 1999) for clusters of significant voxels. The exceedance mass has the advantage of taking account of the *t*-value at each voxel. This mass was calculated by summing all the *t*-values for significant voxels. Since the whole brain beamformer and permutation statistical analysis revealed significant voxels in the primary somatosensory cortex and SFG, we only calculated the exceedance mass for these locations.

## Results

We performed a whole brain MEG imaging and beamforming analysis which revealed power decreases in only the primary somatosensory cortex (beta frequency band) and the SFG (beta and gamma bands), when contrasting the needle manipulation period with the baseline period (*needle manipulation minus baseline* contrast). There were no voxels with significant power increases in the theta, alpha, beta, or gamma frequency bands for this contrast in any brain area (*P* > 0.01 corrected).

### Primary somatosensory cortex

**Figure 1:**
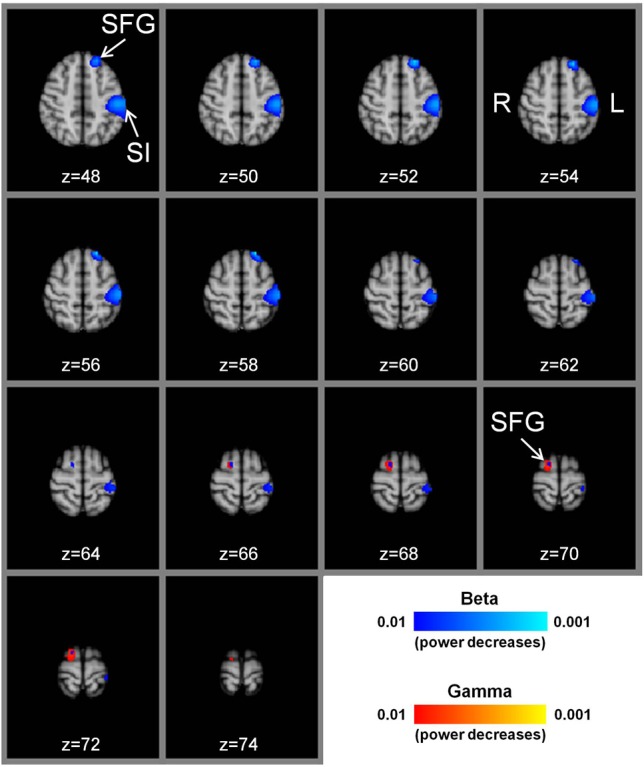
**Group t-statistic maps of power reductions in beta and gamma frequency bands with manual acupuncture needling.** There are prominent power decreases in the beta frequency band (*P* < 0.01) in the contralateral left primary somatosensory cortex for the contrast, *needle manipulation minus baseline*, (needling at acupoint LI-4 in the right hand). There are also significant beta band power decreases in the left superior frontal gyrus (*P* < 0.01). In the ipsilateral right hemisphere, significant beta and gamma band power decreases are located in the superior frontal gyrus (*P* < 0.01). The sequential axial MNI152 brain slices are presented in radiological format with the z axis coordinate (mm). SI = primary somatosensory cortex, SFG = superior frontal gyrus, L = left hemisphere, R = right hemisphere.

The most prominent effect observed with the whole brain beamforming analysis for the contrast, right hand LI-4 *needle manipulation minus baseline*, was a decrease in beta band (13–30 Hz) power in the contralateral left primary somatosensory cortex (Figure 1). The voxel with the highest beta power decrease (*t*-value = −7.8) was located at *x* = −50, *y* = −26, *z* = 52 (MNI152 standard brain coordinates). The exceedance mass for the 1333 significant voxels showing beta band power decreases (*P* < 0.01) in the left primary somatosensory cortex was 8904. There were no significant beta power decreases in the ipsilateral right primary somatosensory cortex (*P* > 0.01). For theta (4–8 Hz), alpha (8–13 Hz), and gamma (30–100 Hz) bands, no significant power decreases were seen in the left or right primary somatosensory cortices (*P* > 0.01).

### Superior frontal gyrus (SFG)

The whole brain MEG analysis also showed reductions in beta band power in the left SFG for right hand LI-4 *needle manipulation minus baseline* (Figure 1). The voxel with the highest beta power decrease (*t*-value = –7.7) was located at *x* = −20, *y* = 38, *z* = 48, and the exceedance mass for the 230 significant voxels (*P* < 0.01) was 1548. For theta, alpha, and gamma bands, there were no significant power decreases in the left SFG (*P* > 0.01). In the right cerebral hemisphere, significant power decreases (*P* < 0.01) were obtained for the beta band (peak voxel located at *x* = 36, *y* = 65, *z* = 70, *t* = −6.1; 32 voxels with exceedance mass = 192) and the gamma band (peak voxel located at *x* = 16, *y* = 0, *z* = 72, *t* = −5.5; 135 voxels with exceedance mass = 710), but not for the theta and alpha bands (Figure 1).

## Discussion

Our study has shown that it is possible to obtain an estimate of neuronal location and oscillatory dynamics using MEG neuroimaging with beamforming analysis despite the absence of precise onset timings when using manual acupuncture needle manipulations. The most prominent finding in the whole brain beamformer analysis was a decrease in power of the beta frequency band in the contralateral primary somatosensory cortex to the contrast, *needle manipulation minus baseline*. In addition, there was a decrease in beta band power in the SFG. In the ipsilateral cerebral hemisphere, power decreases in the beta and gamma bands were only located in the SFG.

### Primary somatosensory cortex

There are many fMRI studies which have applied manual acupuncture needling manipulations in a block design, a paradigm which enables routine and validated analyses using general linear modeling (Fang et al., 2004, 2009; Hui et al., 2005; Napadow et al., 2005; MacPherson et al., 2008; Asghar et al., 2010). In contrast, MEG investigations which have utilized manual acupuncture needling have been limited to one study within MEG sensor space (You et al., 2011), and another which used minimum norm estimates for source reconstruction (Cheng et al., 2011). The main reason for a lack of MEG studies of manual acupuncture are the difficulties associated with subsequent data analysis as the onset times for each needle manipulation stimulus is not accurately measurable. Instead, MEG investigations have made use of electroacupuncture or acupressure where each stimulus onset time is known, with responses being time and phase-locked to the presenting stimulus (Dhond et al., 2008; Witzel et al., 2011). In such MEG studies, equivalent current dipole analysis or a distributed source analysis (minimum norm estimation) has revealed neuronal sources localized only to the contralateral primary somatosensory cortex with either electroacupuncture (Dhond et al., 2008; Witzel et al., 2011) or acupressure (Witzel et al., 2011). The primary somatosensory cortex location was also revealed with manual acupuncture in a MEG study using minimum norm estimation (Cheng et al., 2011). In agreement with these MEG studies, our results also show a similar mapping onto the contralateral primary somatosensory cortex and indicate the useful applicability of beamforming source localization analysis methods in MEG investigations of manual acupuncture.

In the current investigation, there was a prominent decrease in power in the beta frequency band within the contralateral primary somatosensory cortex with manual needle manipulation. This finding is similar to the MEG beta event related desynchronizations reported with electroacupuncture and acupressure in the contralateral primary somatosensory cortex (Witzel et al., 2011), which may be indicative of a common oscillatory response with different types of acupuncture presentation. In the present study we did not seek to determine the finer temporal analysis of the power changes as would be revealed by a time-frequency analysis due to the absence of onset times for each manual acupuncture needle rotation. Without such timings the statistical analysis and interpretation of time-frequency plots is not feasible. When stimulus onset timings are discernible as with electroacupuncture or electronically initiated acupressure, a time-frequency analysis has shown a significant reduction in beta event related desynchronizations over a 15-min period with electroacupuncture but not with acupressure (Witzel et al., 2011). If a methodology could successfully be developed to measure the precise onset times for each manual acupuncture needle rotation and the pre-rotation needle periods discernible, a time-frequency analysis would be able to determine if there are any time-dependent modifications in the beta power reduction.

It remains to be established what the significance is of an acupuncture-induced decrease in the beta band power or whether this might contribute as an underlying mechanism for acupuncture efficacy. In a MEG study, oscillatory power increases in the beta band within the primary somatosensory cortex were reported to visceral pain stimuli and suggested to play a role in the sensory and attentional components of pain processing (Worthen et al., 2011). In a model of human intracutaneous pain, increases in early beta band oscillations were found in MEG sensors overlying sensorimotor areas (Senkowski et al., 2011). EEG studies have reported increases in beta rhythms with tonic pain (Chang et al., 2002; Huber et al., 2006) and functional beta band coupling of sensory and motor cortices in patients with chronic pain (Lalo et al., 2007). Moreover in patients with chronic neurogenic pain, EEG beta overactivations were localized to multiple pain-related areas including the primary somatosensory cortex (Stern et al., 2006). Given these reported modifications of beta oscillations in the processing of noxious signals, and our finding that manual acupuncture can reduce beta power, it would be of value to determine in a future MEG investigation whether manual acupuncture could modulate pain-induced power changes in the beta frequency band in the primary somatosensory cortex.

In the current study using manual acupuncture no significant theta, alpha or gamma frequency band power increases or decreases were seen in the primary somatosensory cortex. In contrast, there were early onset event related synchronizations in theta and gamma frequency bands and prolonged event related desynchronizations in the alpha band with electroacupuncture and acupressure in the primary somatosensory cortex (Witzel et al., 2011). These differences between the MEG studies may be attributable to the type of acupuncture stimuli used or it may reflect differences between the spatial filter algorithms applied. It is possible that we may find theta, alpha, and gamma power modulations if evoked activity (phased locked to the stimulus) could have been considered in our beamforming analysis but this could only be tested by developing an accurate system to measure stimulus onset times for each needle manipulation during manual acupuncture. Oscillations within slower frequency bands may also be of relevance in manual acupuncture, as a wavelet-based time-frequency analysis in MEG sensor space reported delta band (0.5–4 Hz) power increases in the contralateral temporal regions but power decreases in the ipsilateral hemisphere (You et al., 2011).

### Superior frontal gyrus

Our beamformer analysis also localized beta band power decreases with manual acupuncture manipulations to the bilateral SFG (contralateral > ipsilateral), and a gamma band power decrease on the ipsilateral side. Although MEG studies using electroacupuncture or acupressure have only reported localizations to the somatosensory cortex (Dhond et al., 2008; Witzel et al., 2011), an MEG investigation with manual acupuncture had source locations evident in the superior frontal brain areas (Cheng et al., 2011). Neuroimaging fMRI studies of acupuncture have reported SFG activations with manual (Yoo et al., 2004) and laser (Quah-Smith et al., 2010; Hsieh et al., 2011) acupuncture, and deactivations with electroacupuncture (Kong et al., 2007) and manual acupuncture (Yan et al., 2005). Neither these fMRI findings nor our current MEG results are able to indicate what might be the function of the modifications in SFG activity following acupuncture. Using covert versus overt placebo acupuncture needling, the BOLD signal increases in the SFG were suggested to have a role in mediating the expectation toward acupuncture (Chae et al., 2009). Using PET imaging, expectancy effects have been implicated in the response to acupuncture as real or placebo acupuncture both result in activations in the dorsolateral prefrontal cortex when patients expected a “real” acupuncture treatment (Pariente et al., 2005). The SFG has been implicated in a number of roles including processing of pain (Fulbright et al., 2001; Symonds et al., 2006; Tseng et al., 2010), pain mismatch (Ploghaus et al., 2000), response anticipation with increased gamma band power (Fan et al., 2007), attention (Simpson et al., 2011), task control/selection of action sets (Rushworth et al., 2004), and integration in mental imagery (de Borst et al., 2012). Although speculative and requiring future investigation, it is possible that some of these roles associated with the SFG might be of relevance to manual acupuncture.

### MEG studies of sham acupuncture

Although there is ongoing debate and discussion as to what actually constitutes appropriate and credible sham manual acupuncture, it would be desirable in a future MEG investigation to determine and compare the effect on neuronal power of a tactile stimulus (skin tapping), manual acupuncture at non-acupoints, and a retractable acupuncture needle (Streitberger and Kleinhenz, 1998; White et al., 2003). MEG studies using acupressure (Witzel et al., 2011) or tactile stimuli (Gaetz and Cheyne, 2006) have reported beta band cortical event related desynchronizations in the primary somatosensory cortex which could indicate that the beta power reductions in the present investigation may not be specific only to manual acupuncture needling. However, the reductions in beta band power also in the SFG with manual acupuncture needling may be indicative that the overall brain oscillatory responses are different between manual acupuncture needling and acupressure/tactile stimuli.

### Clinical MEG and acupuncture

Studies using fMRI have shown that acupuncture-induced alterations in the BOLD response are modified in patients with clinical conditions indicating a possible role for cortical plasticity. For example, there was a reduction in sensorimotor hyperactivation in carpal tunnel syndrome following clinical acupuncture treatment (Napadow et al., 2007). In stroke patients, acupuncture stimulation produced greater activations in the somatosensory cortex compared to controls (Li et al., 2006), and in post-stroke aphasia patients acupoint stimulation increased activity in language-related brain areas on the lesion side (Li and Yang, 2011). Such fMRI studies are not able to determine if there are any modifications in the dynamics of neuronal rhythms. It would be of particular interest to extend our MEG beamformer study of manual acupuncture in healthy participants, to various patient groupings with clinical conditions and determine whether there are any differences in source location mappings and associated neuronal oscillatory dynamics.

## Conclusion

In conclusion, our results indicate that beamforming can successfully be exploited as an analytical tool for use in MEG investigations of manual acupuncture despite the absence of time-locked needle manipulations. The beamformer was able to reconstruct underlying neuronal source locations mainly in the primary somatosensory cortex (beta band power decreases), and also in the SFG (decreases in beta and gamma band power). Future MEG investigations are needed to determine whether these power changes might contribute toward an underlying mechanism of acupuncture.

## Acknowledgments and funding

We would like to thank all the study participants and the support received from staff and colleagues at the York Neuroimaging Centre. We thank Dr Mark Lythgoe, UCL Institute of Child Health, University College London, for his helpful advice and suggestions. Hugh MacPherson is supported by a National Institute for Health Research funded Career Scientist Award, grant number PAS/03/07/CSA/008.

### Conflict of interest statement

The authors declare that the research was conducted in the absence of any commercial or financial relationships that could be construed as a potential conflict of interest.
